# A New Short Oligonucleotide-Based Strategy for the Precursor-Specific Regulation of microRNA Processing by Dicer

**DOI:** 10.1371/journal.pone.0077703

**Published:** 2013-10-29

**Authors:** Anna Kurzynska-Kokorniak, Natalia Koralewska, Agata Tyczewska, Tomasz Twardowski, Marek Figlerowicz

**Affiliations:** 1 Institute of Bioorganic Chemistry, Polish Academy of Sciences, Poznan, Poland; 2 Institute of Computing Science, Poznan University of Technology, Poznan, Poland; IPMC, CNRS UMR 7275 UNS, France

## Abstract

The precise regulation of microRNA (miRNA) biogenesis seems to be critically important for the proper functioning of all eukaryotic organisms. Even small changes in the levels of specific miRNAs can initiate pathological processes, including carcinogenesis. Accordingly, there is a great need to develop effective methods for the regulation of miRNA biogenesis and activity. In this study, we focused on the final step of miRNA biogenesis; i.e., miRNA processing by Dicer. To test our hypothesis that RNA molecules can function not only as Dicer substrates but also as Dicer regulators, we previously identified by SELEX a pool of RNA oligomers that bind to human Dicer. We found that certain of these RNA oligomers could selectively inhibit the formation of specific miRNAs. Here, we show that these specific inhibitors can simultaneously bind both Dicer and pre-miRNAs. These bifunctional riboregulators interfere with miRNA maturation by affecting pre-miRNA structure and sequestering Dicer. Based on these observations, we designed a set of short oligomers (12 nucleotides long) that were capable of influencing pre-miRNA processing *in vitro,* both in reactions involving recombinant human Dicer and in cytosolic extracts. We propose that the same strategy may be used to develop effective and selective regulators to control the production of any miRNA. Overall, our findings indicate that the interactions between pre-miRNAs and other RNAs may form very complex regulatory networks that modulate miRNA biogenesis and consequently gene expression.

## Introduction

Recently, it has become increasingly clear that the majority of human protein-coding genes are regulated by microRNAs (miRNAs) [1], [2]. It means that miRNAs are involved in many biological processes, including developmental timing, growth, differentiation and apoptosis [3], [4], [5]. Furthermore, there are several lines of evidence indicating that miRNAs also participate in host-virus interactions [6], [7], [8]. Thus, the accurate control of individual miRNA biogenesis is critical for the functions of numerous living organisms, including humans. Even small changes in miRNA production and accumulation can initiate pathological processes, e.g., carcinogenesis, neurodegeneration, or immune system or rheumatic disorders [9], [10], [11], [12].

The ribonuclease Dicer is one of the key enzymes involved in the biogenesis of miRNAs in humans. This enzyme excises mature, functional miRNAs from 50–70 nt stem-loop precursors called pre-miRNAs [13]. Human Dicer is a 220-kDa multidomain protein comprising a putative helicase domain (homologous to DExD/H-box helicases), a DUF283 domain (domain of unknown function), a PAZ (Piwi-Argonaute-Zwille) domain, two RNase III domains, and a double-stranded RNA (dsRNA) binding domain (dsRBD) [13], [14], [15], [16], [17]. To date, only one Dicer gene (*DICER1*) has been identified in each analyzed mammal genome. There are numerous reports indicating that the expression of this gene is highly regulated at many different levels; consequently, the pattern of Dicer accumulation varies substantially among tissues or cell types depending on their function and stage of development. The human Dicer gene has a complex structure. Its open reading frame contains 26 exons. In addition to the protein-coding exons, the Dicer gene contains a number of non-protein-coding exons that compose the 5′ and 3′ untranslated regions (UTRs). For example, there are three variants of non-protein coding exon 1 (5′-UTR exon 1a–c). Further, various 5′-UTRs with different *cis* RNA regulatory elements can be produced via alternative splicing. The ATG start codon is localized in exon 2 [18]. To date, four mRNA variants encoding full-length Dicer enzymes have been identified (http://www.ensembl.org/Homo_sapiens/Transcript/Summary?db=coreg=ENSG00000100697r=14∶95552565–95624347;t = ENST00000393063). In addition, numerous shorter alternative splicing products have been found. Some of these products encode proteins that retain only the functional RNase III domains of Dicer, and some variants do not encode any protein [19], [20].

The polymorphism of the 5′-UTR was reported to have profound effects on the translational efficiency of Dicer mRNA. Furthermore, the exon 1 variants exhibit very restricted patterns of tissue distribution [18]. The 3′-UTR is also involved in the regulation of Dicer gene expression. This region can be targeted by several miRNAs, for instance, miR-103/107 [20], [21] or let-7 miRNA [22]. Interestingly, target sequences for let-7 were also found in the coding region of Dicer mRNA [22]. Further, Wiesen et al. demonstrated that the level of cellular Dicer mRNA is frequently not correlated with the level of Dicer protein. Based on these observations, the authors concluded that the regulation of Dicer expression occurs mainly at the post-transcriptional level [23]. In addition, the mechanisms that regulate Dicer production appear to be fairly sophisticated. For instance, in particular subtypes of breast cancer, different isoforms of Dicer mRNA are expressed. Most of these isoforms have truncated 5′- and 3′-UTRs, while their coding regions are unchanged. Transcripts that lack the large fragments of the 3′-UTR cannot be targeted by several miRNAs. Thus, the regulation of Dicer expression by at least some of its regulatory miRNAs is lost in these cells [19], [20]. There is also no clear correlation between the level of Dicer accumulation and cancer progression. It is known that the reduced expression of Dicer may be associated with poor prognosis in some types of lung cancers [24]. In contrast, increases in the expression of this enzyme have been detected in prostate cancer and Burkitt’s lymphoma cells [25], [26]. Moreover, specific changes in Dicer expression are associated with different stages of particular tumors. A transient upregulation of Dicer gene expression is observed in the early stages of peripheral adenocarcinoma of the lung, whereas Dicer is downregulated in more advanced stages of this cancer [27].

Recently, several factors regulating the expression of Dicer gene and the activity of the Dicer protein have been identified. Wiesen et al. demonstrated that the expression of the Dicer gene can be repressed by stress (induced by reactive oxygen species and phorbol esters) or by Ras oncogene activation [23]. These authors also showed that at the protein level, Dicer expression may be downregulated by approximately 4000 base pair-long double-stranded RNAs and interferon alpha (type I interferon) and upregulated by interferon gamma (type II interferon). It has also been reported that the activity of the Dicer protein can be inhibited by certain cytoplasmic factors. These inhibitors have not yet been defined; however, it has been shown that the addition of cytosolic extracts from HeLa cells inhibited the efficient processing of pre-miR-138 by Dicer *in vitro*. Interestingly, under the same conditions, the maturation of other tested miRNAs was not affected [28]. The authors suggested that Dicer may compete with undefined inhibitors for binding with specific pre-miRNAs. A similar competitive inhibition mechanism was proposed by Viswanathan et al. [29], [30]. They showed that Lin28, a highly conserved RNA-binding protein, can selectively block the processing of the let-7 pre-microRNA by Dicer through specific binding to the precursor. In contrast, the KH-type splicing regulatory protein (KSRP) can promote the production of certain miRNAs by binding to the loop region of their precursors [31].

We have attempted to determine whether the activity of human Dicer can be regulated not only by proteins but also by RNA molecules. Recently, we demonstrated that recombinant human Dicer (hDicer) activity can be modulated by the binding of RNA molecules to this enzyme [32]. In addition, we identified RNA oligomers that differentially affected the processing of various pre-miRNAs. Among the tested oligomers, we identified typical competitive, allosteric and mixed inhibitors. Here, we describe short RNA molecules that can selectively regulate miRNA production by targeting both pre-miRNA and hDicer.

## Results

### Selective Inhibition of miRNA Processing by hdicer

**Figure 1:**
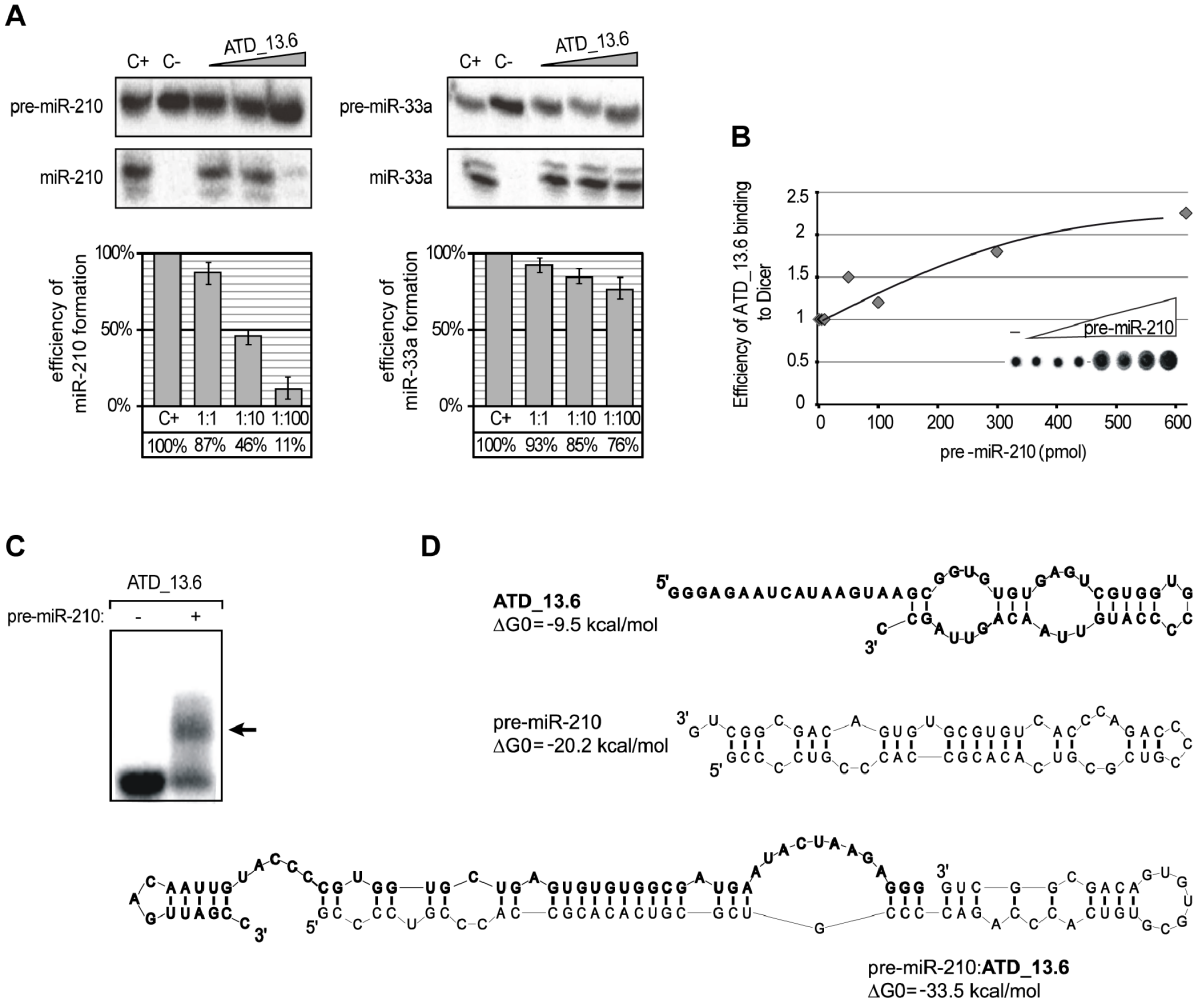
ATD_13.6 selectively binds to pre-miR-210 and inhibits its processing by hDicer. (**A**) Radiolabeled pre-miR-210 or pre-miR-33a was incubated with hDicer in the presence of ATD_13.6. Control reactions lacked ATD_13.6 (C+) or ATD_13.6 and the enzyme (C−). The triangles indicate increasing amounts of ATD_13.6 (applied pre-miRNA and oligomer molar ratios: 1∶1, 1∶10, 1∶100). The diagrams show the average efficiency of miRNA production from three independent experiments; error bars represent the standard deviations. (**B**) Plot representing the positive correlation between increasing pre-miR-210 concentration and the efficiency of ATD_13.6 binding to hDicer. In the experiment shown, equimolar concentrations of Dicer and radiolabeled ATD_13.6, and increasing amounts of pre-miR-210 were used (molar ratios from 1∶1 to 1∶600). The control reaction lacked the pre-miRNA (−). All reaction mixtures were simultaneously filtered on nitrocellulose membranes (shown in the plot). (**C**) The binding of ATD_13.6 to pre-miR-210. Radiolabeled ATD_13.6 was denatured and renatured alone (−) or in the presence of pre-miR-210 (+). The reactions were separated in a native polyacrylamide gel. The position of the ATD_13.6 and pre-miR-210 complex is indicated by the arrow. (**D**) The predicted secondary structures of ATD_13.6 (bold), pre-miR-210 (regular) and a complex formed by the two molecules. The ΔG0 values, expressed in kcal/mol, are shown next to the structures.

**Figure 2:**
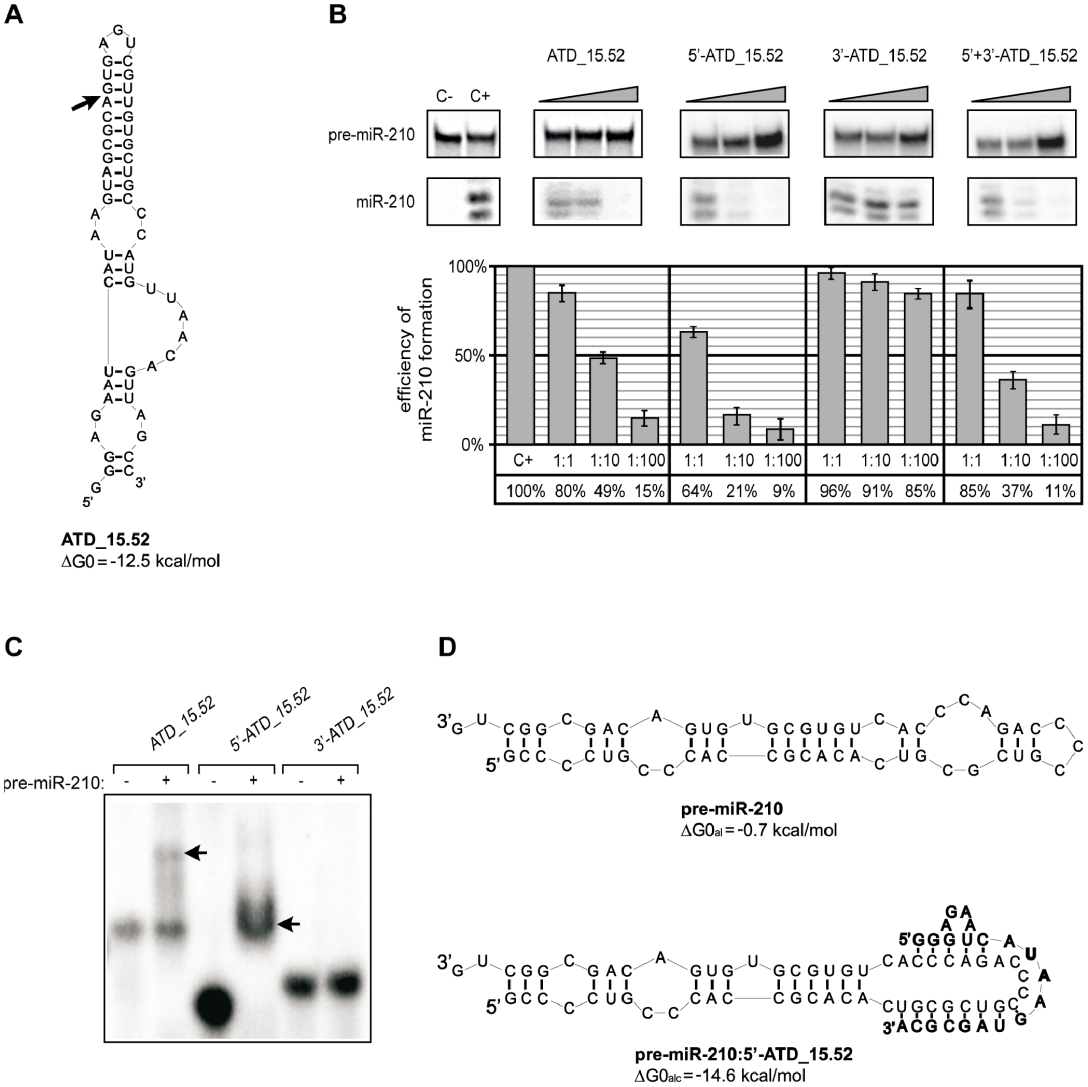
ATD_15.52 is cut by hDicer, and the cleavage product functions as a specific inhibitor. (**A**) Predicted secondary structure of ATD_15.52. The ΔG0 value, expressed in kcal/mol, is shown next to the structure. The arrow indicates the determined hDicer cleavage site. (**B**) Influence of ATD_15.52 and its two hDicer cleavage fragments on the efficiency of miR-210 production. Radiolabeled pre-miR-210 was incubated with hDicer in the presence of ATD_15.52, one of its fragments (5′- or 3′-ATD_15.52) or both fragments (5′+3′ ATD_15.52). Control reactions lacked the enzyme and oligomers (C−) or oligomers only (C+). Triangles indicate increasing concentrations of applied oligomers (pre-miRNA:oligomer molar ratios of 1∶1, 1∶10, and 1∶100). The diagrams show the average efficiency of miR-210 production based on three independent experiments; error bars represent the standard deviations. (**C**) Binding of ATD_15.52 and its fragments to pre-miR-210. Radiolabeled ATD_15.52 or one of its fragments (5′-ATD_15.52 or 3′-ATD_15.52) was denatured and renatured alone (−) or in the presence of pre-miR-210 (+). The reactions were separated in a native polyacrylamide gel. The positions of the complexes formed by ATD_15.52 or its 5′-fragment and pre-miR-210 are indicated by arrows. (**D**) The predicted secondary structures of pre-miR-210 (regular) and its complex with the 5′ fragment of ATD_15.52 (bold). The free energies calculated for the apical fragment of pre-miR-210 (ΔG0_al_) or for the complex formed by the corresponding apical fragment of pre-miR-210 and 5′-ATD_15.52 (ΔG0_alc_) are shown next to the structures.

Previously, we showed that RNA oligomers can bind to hDicer and regulate its activity. In a pool of hDicer-binding RNA oligomers identified by SELEX (using approximately 56-nt long oligoribonucleotides), we found molecules that inhibited pre-miRNA processing by acting as: (i) competitive inhibitors – these oligomers were cut by hDicer; and (ii) allosteric inhibitors – these molecules were not digested by hDicer [32]. Interestingly, among the selected oligomers, ATD_13.6 (Fig. 1) and ATD_15.52 (Fig. 2) selectively and permanently repressed the excision of miR-210 from its precursor. Our preliminary observations suggested that ATD_13.6 should be classified as allosteric inhibitor and that ATD_15.52 should be considered a competitive inhibitor [32]. Unfortunately, our earlier experiments did not reveal any molecular determinants of inhibitor specificity. To identify these determinants, both oligomers were subjected to more detailed studies.

Our previous experiments demonstrated that ATD_13.6 efficiently inhibits pre-miR-210 cleavage and only slightly affects pre-miR-33a processing (Fig. 1A). This oligonucleotide forms a stable complex with hDicer (Fig. S1A), but it is not cut by the enzyme [32]. To gain a better understanding of the nature of ATD_13.6:Dicer interactions, we performed a standard competitive displacement assay. A set of reaction mixtures containing the same amounts of radiolabeled ATD_13.6 (100 nM) and hDicer, and gradually increasing amounts of the substrate pre-miR-210 (from 0 to 60 µM) were prepared. After one hour of incubation, the reaction mixtures were filtered through a nitrocellulose membrane, and the radioactivity retained on the filter was quantified. Surprisingly, we observed that ATD_13.6 binding to hDicer was enhanced by the substrate in a concentration-dependent manner (Fig. 1B). The obtained results suggested that ATD_13.6 might interact not only with hDicer but also with pre-miR-210. To test this assumption, we analyzed ATD_13.6:pre-miR-210 binding using an electrophoretic mobility shift assay (EMSA). In this assay, a 100-fold molar excess of pre-miR-210 (10 µM) to radiolabeled ATD_13.6 was used. The data obtained revealed that pre-miR-210 and ATD_13.6 indeed form a complex (Fig. 1C). The secondary structure of this complex, as predicted by the program RNAstructure, was thermodynamically more stable than the structures adopted by its individual components (Fig. 1D). To test whether the selective inhibition of pre-miR-210 cleavage by ATD_13.6 results from the specific interactions between these two molecules, we performed control reactions involving several pre-miRNA species, i.e., pre-miR-16-1, -21, and -33a. The obtained results confirmed that ATD_13.6 formed complexes only with pre-miR-210. We did not observe interactions between ATD_13.6 and pre-miR-16-1, -21, and -33a even when their high molar excess was applied (Fig. S2A). In addition, we tested the effects of increasing concentrations of pre-miR-16-1, -21, -33a, and -210 (1, 10, and 100 pmoles) on ATD_13.6 binding to hDicer. As expected, we found that pre-miR-16-1, -21, and -33a displaced ATD_13.6 from binding to the enzyme (Fig. S3). The most profound effect was observed for pre-miR-16-1. These findings suggest that the applied pre-miRNAs outcompeted ATD_13.6 for binding to hDicer. Exclusively for one reaction in which pre-miR-210 was added to the ATD_13.6 and hDicer, another slowly migrating complex was formed. This observation indicates that the three molecules: pre-miR-210, ATD_13.6 and hDicer may mutually interact.

Our earlier studies also showed that ATD_15.52, another oligomer that specifically repressed miR-210 production, was bound by hDicer (Fig. S1B) and cut into two fragments: a 21-nt long 5′-fragment (5′-ATD_15.52) and a 35-nt long 3′-fragment (3′-ATD_15.52) (Fig. 2A). We hypothesized that one or both products of ATD_15.52 digestion might interfere with hDicer activity. To test this hypothesis, we investigated how the addition of 5′-ATD_15.52, 3′-ATD_15.52 or both fragments influenced the ability of hDicer to cut pre-miR-210. The effects of ATD_15.52 and its fragments on hDicer activity were tested *in vitro* in standard pre-miRNA digestion reactions. The concentrations of the radiolabeled pre-miR-210 and of the enzyme were the same in all samples; only the amount of the oligomer was altered. In each series, the oligomer:hDicer molar ratios of 1∶1, 10∶1, and 100∶1 were tested. In addition, two control reactions were always run, namely, a reaction without the oligomer and a reaction without hDicer. All samples were incubated at 37°C for 30 minutes. The amount of the 5′-terminally labeled pre-miR-210 and its cleavage products was determined for each reaction, and the efficiency of miR-210 production in the presence or absence of the oligomer (i.e., the oligomers’ capacity to inhibit the digestion of pre-miR-210 by hDicer) was calculated. The influence of the oligomers on miRNA production was expressed as a percentage, with 100% defined as the miRNA production in reactions conducted without the oligomer (Fig. 2B).

We found that the production of miR-210 was significantly reduced after the addition of 5′-ATD_15.52 (to approximately 10%). The inhibition of pre-miR-210 cleavage was also observed when the full-length oligomer and both its fragments were added to the reaction mixture. Only residual inhibition was detected when 3′-ATD_15.52 was added (Fig. 2B). These results indicated that the 5′-product of ATD_15.52 cleavage by hDicer alone can effectively inhibit the processing of pre-miR-210. To learn more about the mechanism underlying this process, we examined whether 5′-ATD_15.52 could interact with pre-miR-210. The results of the EMSA experiment involving radiolabeled ATD_15.52 or its fragments and pre-miR-210 revealed that 5′-ATD_15.52 forms a very stable complex with pre-miR-210 (Fig. 2C). ATD_15.52 was also capable of forming a complex with pre-miR-210, albeit less effectively than its 5′ fragment. The 3′ fragment did not interact with the miR-210 precursor. The prediction of the secondary structure of the pre-miR-210∶5′-ATD_15.52 complex using the RNAstructure program showed that 12–13 nucleotides of the 5′ fragment can base-pair with the apical fragment of pre-miR-210. Consequently, the native structure of pre-miR-210 is disrupted (Fig. 2D). Again, to prove that the selective inhibition of pre-miR-210 cleavage by ATD_15.52 (precisely by its 5′ fragment) results from the specific interactions between the two molecules, we performed control reactions involving several pre-miRNA species, i.e., pre-miR-16-1, -21, and -33a (Fig. S2B). According to our expectations, we did not observe interactions between ATD_15.52 and pre-miR-16-1, -21, and -33a even when their high molar excess was applied.

### RNA Oligomers Targeting Pre-miRNAs may Control miRNA Production

**Figure 3:**
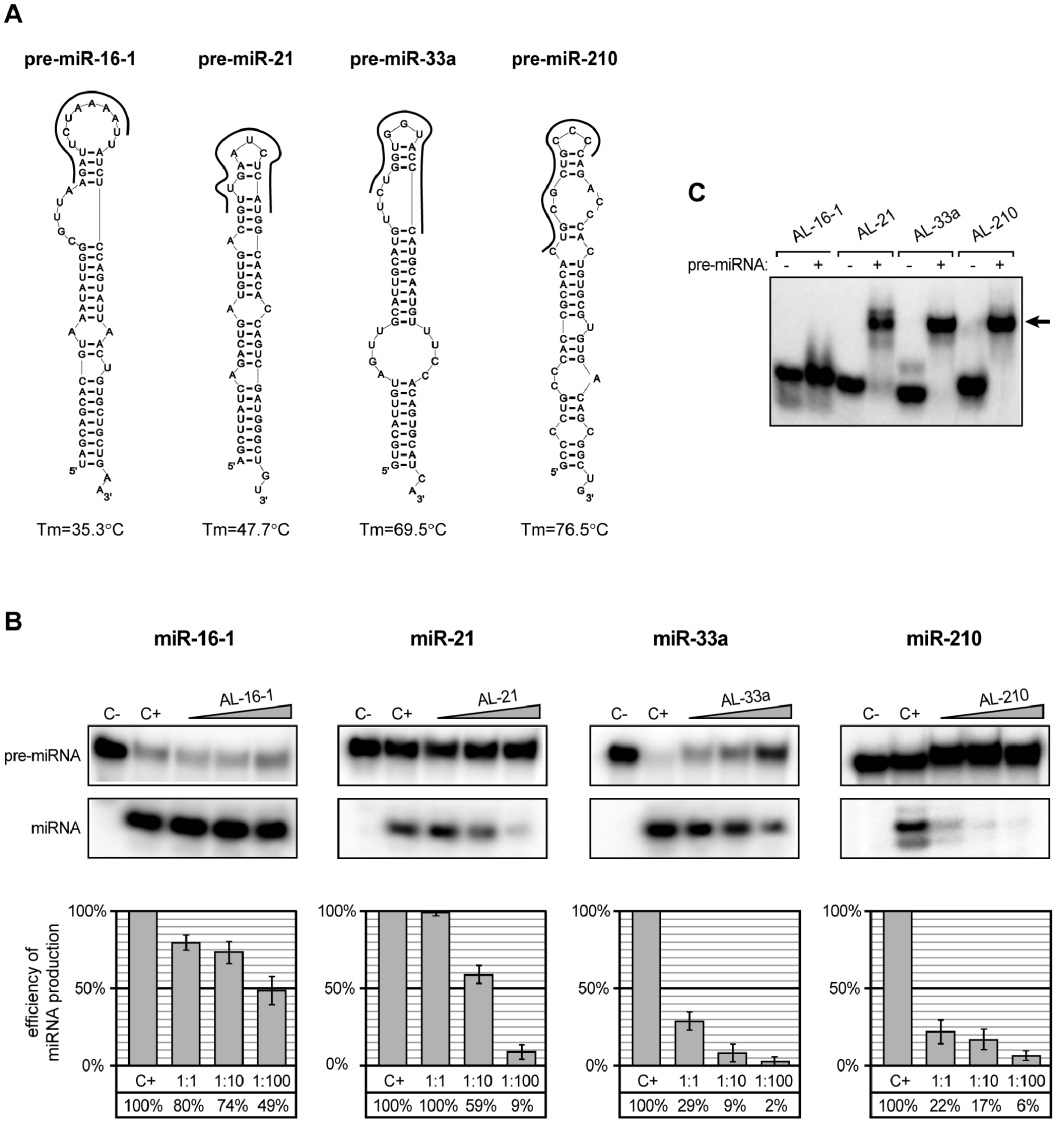
Twelve-nucleotide oligomers targeting apical fragments of pre-miRNA hairpins disturb precursor processing by hDicer. (**A**) The predicted secondary structures of four tested pre-miRNAs (pre-miR-16-1, pre-miR-21, pre-miR-33a and pre-miR-210). Oligonucleotides are shown as thick, black curves. The calculated melting temperatures of duplexes formed by nucleotides present in the apical fragments of each pre-miRNA and corresponding oligomer are shown below the structures. (**B**) Radiolabeled pre-miRNAs were incubated with hDicer in the presence of the appropriate 12-nt oligomers. Control reactions lacked the enzyme and oligomer (C−) or the oligomer only (C+). Triangles represent increasing amounts of indicated oligomers (pre-miRNA:oligomer molar ratios of 1∶1, 1∶10, and 1∶100). The diagrams show the average efficiency of miRNA production based on three independent experiments; error bars represent the standard deviations. (**C**) Binding of the 12-nt oligomers to the corresponding pre-miRNAs. Radiolabeled oligomers were denatured and renatured alone (−) or in the presence of the corresponding pre-miRNAs (+). Reactions were separated in a native polyacrylamide gel. The position of complexes formed by oligomers and pre-miRNAs are indicated with arrows.

The above results clearly demonstrated that interactions between pre-miR-210 and selected RNA oligomers can influence the efficiency and/or pattern of precursor digestion by hDicer. Furthermore, the data collected suggested that oligomers as short as 12 nt can inhibit pre-miRNA processing by interacting with the apical fragment of the hairpin structure adopted by this precursor. To determine whether these observations are specific cases of a more general rule, we tested the effects of four 12-nt oligomers, AL-16-1, AL-21, AL-33a and AL-210, on pre-miR-16-1, pre-miR-21, pre-miR-33a and pre-miR-210 processing. Each oligomer was complementary to the apical fragments of the hairpin that could be formed by the corresponding miRNA precursors (Fig. 3A). For each pair of pre-miRNA and complementary oligomer, five reactions were performed. The three experimental reaction mixtures contained hDicer, pre-miRNA and oligomer; the molar ratios of pre-miRNA to oligomer were 1∶1, 1∶10 and 1∶100. The control reactions lacked either hDicer (C−) or the oligomer (C+). For the pre-miR-33a:AL-33a and pre-miR-210:AL-210 pairs, very effective inhibition was observed, even at a low concentration of the oligomer (Fig. 3B). For the pre-miR-21:AL-21 pair, miR-21 formation was effectively inhibited only when a 100-fold molar excess of oligomer was applied. Finally, one oligomer, AL-16-1, did not efficiently reduce pre-miR-16-1 digestion by hDicer (Fig. 3B). As discussed earlier, each oligomer was complementary to the apical fragment of the hairpin structure adopted by the corresponding pre-miRNA. Thus, all of these oligomers targeted the same region of the precursor, but their capacity to inhibit pre-miRNA processing differed significantly.

To determine whether oligomers were capable of binding to the corresponding pre-miRNAs, we performed standard EMSA experiments. All of these experiments used a 100-fold molar excess of pre-miRNAs to radiolabeled oligomers (Fig. 3C). The obtained results revealed that AL-33a and AL-210 formed stable complexes with pre-miR-33a and pre-miR210, respectively. The complex formed by AL-21 and pre-miR-21 was slightly weaker, and AL-16-1 showed only residual capacity to bind its target. The melting temperatures of the duplexes formed by the complementary apical fragment of each pre-miRNA and the corresponding oligomer (Tm) were calculated using HyTher (http://ozone3.chem.wayne.edu). For the tested oligomers, the Tm changed as follows: 35.3°C (AL-16-1), 47.7°C (AL-21), 69.5°C (AL-33a) and 76.5°C (AL-210). The calculated values were consistent with the results of the EMSA, i.e., a higher Tm corresponded to more stable complexes (Fig. 3A and C). Based on these observations, one can hypothesize that there is a correlation between Tm of the pre-miRNA:oligomer complex and the capacity of the oligomer to inhibit pre-miRNA processing (Fig. 3B).

**Figure 4:**
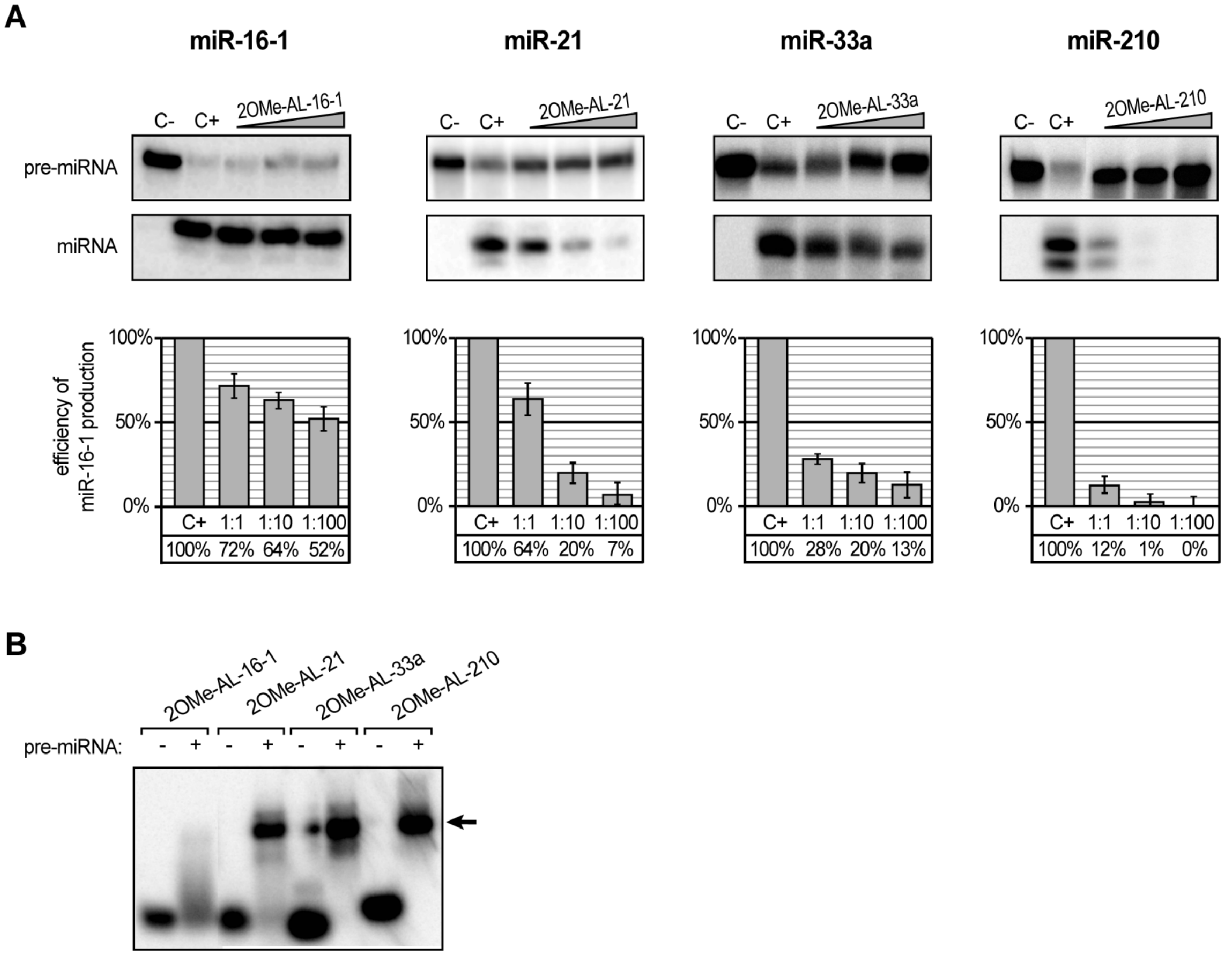
The influence of 12-nucleotide 2′-O-methylated oligomers targeting apical fragments of pre-miRNA hairpins on miRNA production by hDicer. (**A**) Radiolabeled pre-miRNAs were incubated with hDicer and 12-nt 2OMe-oligomers. Control reactions lacked the enzyme and oligomer (C−) or the oligomer only (C+). Triangles represent increasing amounts of the indicated 2OMe-oligomers (pre-miRNA:oligomer molar ratios of 1∶1, 1∶10, and 1∶100). The diagrams show the average efficiency of miRNA production based on three independent experiments; error bars represent the standard deviations. (**B**) Binding of 2OMe-oligomers to the corresponding pre-miRNAs. Radiolabeled oligomers were denatured and renatured alone (−) or in the presence of the corresponding pre-miRNAs (+). The reactions were separated in a native polyacrylamide gel. The positions of the complexes formed by the oligomers and pre-miRNAs are indicated with arrows.

**Figure 5:**
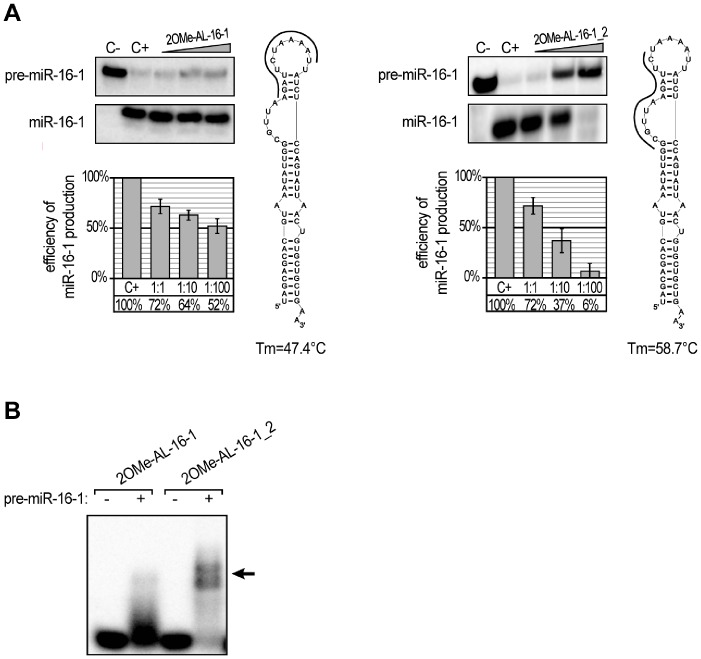
The stability of the complexes formed by pre-miR-16-1 and complementary oligomers determines the efficacy of miR-16-1 production by hDicer. (**A**) Comparison of the efficiency of miR-16-1 production by hDicer in the presence of the two oligomers targeting distinct apical regions of pre-miR-16-1. Radiolabeled pre-miR-16-1 was incubated with hDicer in the presence of either 2OMe-AL-16-1 (*Left panel*) or 2OMe-AL-16-1_2 (*Right panel*). Control reactions lacked the enzyme and oligomer (C−) or the oligomer only (C+). Triangles represent increasing amounts of the indicated 2OMe-oligomers (pre-miR-16-1:oligomer molar ratios of 1∶1, 1∶10, and 1∶100). The diagrams show the average efficiency of miR-16-1 production based on three independent experiments; error bars represent the standard deviations. The predicted secondary structures of pre-miR-16-1 are shown next to the diagrams. Complementary 12-nt 2OMe-oligomers are shown as thick, black curves. The calculated melting temperatures of duplexes formed by nucleotides present in the apical fragments of pre-miR-16-1 and each oligomer are shown below the structures. (**B**) Binding of pre-miR-16-1-specific oligomers to the precursor. Radiolabeled oligomers were denatured and renatured alone (−) or in the presence of pre-miR-16-1 (+). The reactions were separated in a native polyacrylamide gel. The positions of the complexes formed by 2OMe-AL-16-1_2 and pre-miR-16-1 are indicated with arrows.

To test the above hypothesis, we performed a similar set of experiments but replaced the previously used oligomers with their 2′-O-methylated analogues. If Tm is the major factor affecting oligomer inhibition capacity, the application of a modified oligomer should significantly decrease pre-miRNA processing by hDicer. The predicted Tm values of the 2OMe-oligo:pre-miRNA complexes were as follows: 47.4°C for 2OMeAL-16-1, 56.1°C for 2OMe AL-21, 82.0°C for 2OMe AL-33a, and 87.1°C for 2OMe AL-210. The *in vitro* hDicer cleavage assay performed with the modified oligomers revealed that inhibition was enhanced, especially for the reactions containing a 1∶1 molar ratio of modified oligomer to pre-miRNA (Figs. 3B and 4A). The formation of specific complexes was analyzed via EMSA (Fig. 4B), and the obtained results proved that more effective binding of oligonucleotides to pre-miRNAs increases oligomer inhibition capacity. However, the effect observed for 2OMe-AL-16-1 was less profound than we expected based on the calculated Tm. Oligomers AL-16-1 and 2OMe-AL-16-1 similarly affected the formation of miR-16-1, especially at the highest concentration (Figs. 3B and 4A). Further, although the Tm values established for the complexes formed by 2OMe-AL-16-1 and AL-21 were similar (47.4°C and 47.7°C, respectively), the latter inhibited the corresponding miRNA formation more effectively. These observations may indicate that not only Tm but also pre-miRNA accessibility by the oligomer might be especially important. To address this problem, we designed a new 2OMe-oligomer (2OMe-AL-16-1_2) complementary to the other partially single-stranded sequence of the apical fragment of pre-miR-16-1 (Fig. 5A, *Right panel*). An EMSA using pre-miR-16-1 and 2OMe-AL-16-1_2 proved that the new oligomer bound more tightly to pre-miR-16-1 than 2OMe-AL-16-1 (Fig. 5B). The calculated Tm of 2OMe-AL-16-1_2 was 58.7°C, which is over 10°C higher than that of 2OMe-AL-16-1 (Fig. 5A). As expected, 2OMe-AL-16-1_2 was also a more effective inhibitor of pre-miR-16-1 processing than its counterpart 2OMe-AL-16-1.

### Effective and Selective Inhibition of Individual miRNA Production

**Figure 6:**
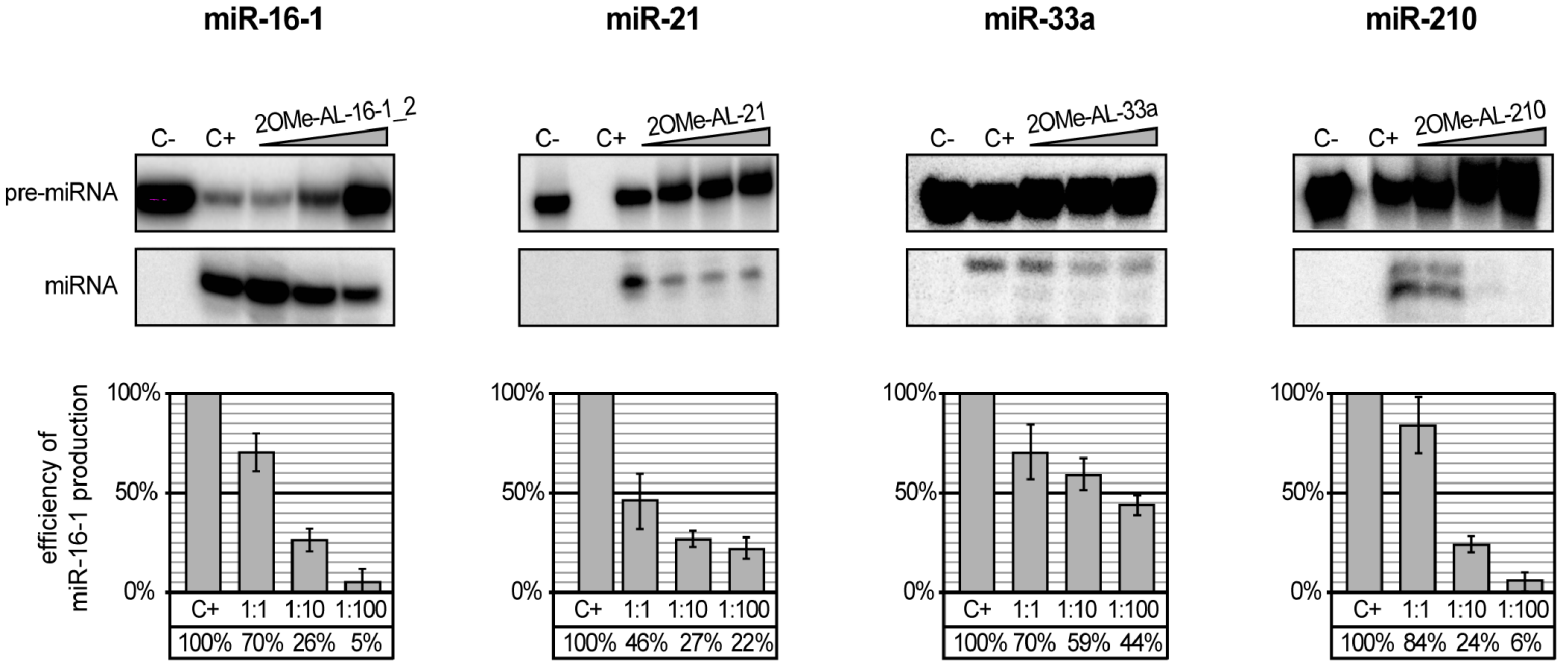
The influence of 12-nucleotide 2′-O-methylated oligomers targeting apical fragments of pre-miRNA hairpins on miRNA production in HeLa cytosolic extracts. Radiolabeled pre-miRNAs were incubated with HeLa cytosolic extracts in the presence of the corresponding 12-nt 2OMe-oligomers. Control reactions lacked the extract and oligomer (C−) or the oligomer only (C+). Triangles represent increasing amounts of the indicated 2OMe-oligomers (pre-miRNA:oligomer molar ratios of 1∶1, 1∶10, and 1∶100). The diagrams show the average efficiency of miRNA production based on three independent experiments; error bars represent the standard deviations.

The experiments described above showed that short oligomers can be used to prevent miRNA formation in a simple tri-component system (Dicer:pre-miRNA:oligomer). The next question we attempted to answer was whether short RNAs are capable of repressing pre-miRNA processing in a more complex system approximating natural conditions. To this end, we tested whether the most potent inhibitors identified in our previous experiments maintained their activities in a cytoplasmic extract from HeLa cells. Cytoplasm was obtained from HeLa cells, and pre-miRNA cleavage experiments were performed as described by Leuschner and Martinez [33]. For each radioactively labeled pre-miRNA and complementary 2OMe-oligomer pair, an identical set of five reactions was carried out. All five samples contained the same amount of radioactively labeled pre-miRNA. Three of the samples contained different molar ratios of pre-miRNA:2OMe-oligomer (1∶1, 1∶10 and 1∶100). One control reaction contained no oligomer, and the other contained only pre-miRNA and the buffer used for cytoplasmic extract preparation. The results of the experiments performed for four pairs, 2OMe-AL-16-1_2:pre-miR-16, 2OMe-AL-21:pre-miR-21, 2OMe-AL-33a:pre-miR-33a, and 2OMe-AL-210:pre-miR-210, are shown (Fig. 6). Almost no miR-16-1 or miR-210 was produced at a 100-fold molar excess of 2OMe-AL-16-1_2 or 2OMe-AL-210. Interestingly, a relatively high but constant level of inhibition of pre-miR-21 processing was observed for every tested concentration of 2OMe-AL-21. The weakest inhibitory effect was exerted by 2OMe-AL-33a. A subsequent series of experiments demonstrated that pre-miRNAs were selectively targeted by complementary oligomers (Fig. S4). For all but one non-matching oligomer, none or some residual inhibition was observed only when a 100-fold molar excess was applied. This exception was 2OMe-AL-210. For this oligomer we observed significant inhibition of any tested pre-miRNA processing by hDicer. Further studies revealed that this precursor-nonspecific inhibition resulted from 2OMe-AL-210 binding to the enzyme (Fig. S5). Nevertheless, the most prominent inhibition of miRNA production by 2OMe-AL-210 was observed for the complementary precursor, pre-miR-210. Altogether, the collected data indicate that oligomers as short as 12 nt can function as effective and selective inhibitors of miRNA production in the cytoplasm.

## Discussion

Dicer or its analogs are indispensable elements of all RNAi pathways. There is no doubt that the precise regulation of these pathways, including the regulation of all involved proteins, is a very complex process. Recently, several potential regulators of Dicer activity have been identified. It was shown that quadruplex-binding compounds (porphyrazines and bisquinolinium compounds) can inhibit Dicer-mediated digestion of guanosine-rich siRNA precursors [34]. Ma et al. reported that a putative helicase domain of Dicer can exert an autoinhibitory effect on this enzyme [35]. Lima et al. demonstrated that human Dicer can interact with different regions of RNA transcripts and bind to both single- and double-stranded RNA molecules [36]. It has also been reported that adenoviruses protect their RNAs by producing a high amount of long self-complementary transcript, which effectively competes for Dicer binding with other molecules. As a result, the remaining viral transcripts are protected from cleavage [37]. Recently, it has been demonstrated that RNA molecules can interfere with miRNA maturation by sequestrating the RNase III Drosha, which is another enzyme engaged in miRNA biogenesis [38]. However, to date there have been no reports showing that RNA molecules can selectively target both miRNA precursors and Dicer and thus regulate individual miRNA maturation. In our studies, we identified RNA oligomers that acted as bifunctional regulators. These oligomers specifically affected miR-210 maturation by binding both Dicer and pre-miR-210 (Figs. 1, 2, 3, S1 and S2). More specifically, in addition to binding Dicer, they acted as *sui generis* antagomiRs, i.e., short RNA molecules that bind to miRNAs and inhibit their proper functioning [39]. In our case, however, we were dealing with antagopre-miRs, i.e., short RNA molecules that interact with pre-miRNAs and prevent miRNA formation.

One of the most extensively studied examples of the selective regulation of pre-miRNA processing involves the RNA-binding protein Lin28 and the let-7 pre-miRNA [29], [30], [40], [41]. Lin28 can sequester pre-let-7 miRNA to prevent Dicer-mediated processing. It was shown that Lin28 binds directly to pre-let-7, presumably through the specific recognition of the let-7 pre-miRNA terminal loop. Piskounova et al. showed that a conserved cytosine residue in the loop of pre-let-7 g is essential for Lin28 binding [42]. In contrast, Heo et al. emphasized the role of a tetra-nucleotide sequence motif (GGAG) present in the terminal loop of pre-let-7 in the selective binding of Lin28 to this precursor [43]. The authors demonstrated that Lin28 recruits a terminal uridylyl transferase (TUTase) to the pre-let-7. This noncanonical polymerase adds an oligouridine tail to the pre-let-7, thereby preventing the modified precursor from being efficiently processed by Dicer. The polyuridylated RNA is subsequently degraded, and consequently, the let-7 miRNA depletion is observed. In contrast, another RNA binding protein, the KH-type splicing regulatory protein KSRP, promotes the maturation of certain pre-miRNAs, e.g., pre-let-7a-1 [31]. Trabucchi et al. found that one of the KSRP domains recognizes a short G-rich stretch present in the terminal loop of pre-let-7a-1a with high specificity and affinity. They also showed that KSRP can function as a component of both the Drosha and Dicer complexes. Moreover, by binding to the apical loops of certain miRNA precursors, KSRP participates in the nucleo-cytoplasmic transport of targeted pre-miRNAs by exportin-5. Based on the immunoprecipitation experiments, the authors designated two pools of miRNA precursors, those associated with processing complexes including KSRP and those associated with complexes lacking KSRP. Interestingly, Michlewski et al. showed that hnRNP A1, the RNA-binding protein involved in many aspects of RNA processing, may bind to the terminal loop of these miRNA precursors for which processing is not affected by KSRP [44]. These authors hypothesized that there is a group of miRNA precursors with conserved apical loops and that their processing strongly depends on the binding of auxiliary factors (such as hnRNP A1) to this conserved structural element. One such specific molecule is a precursor of miR-18a. Michlewski et al. reported that oligonucleotides complementary to the conserved terminal loop of the miR-18a precursor selectively abolished its processing. In contrast, oligonucleotides targeting nonconserved terminal loops of miR-16-1 and -27a precursors did not affect maturation of these miRNAs. The authors suggested that hnRNP A1 is not essential for the processing of miRNA precursors bearing nonconserved terminal loops, such as miR-16-1 and -27a.

The above studies concentrated on proteins that are involved in the regulation of miRNA maturation. Our studies focused on the question of whether RNA molecules can also modulate the processing of individual pre-miRNAs. We did not consider the conservation of the miRNA precursors but rather examined whether the pre-miRNAs exhibited secondary structures that may be accessible for interactions with other RNA molecules. Consistent with the published data, we demonstrated that the terminal loop regions are important for controlling miRNA processing by Dicer. Indeed, this region of miRNA precursors may serve as a binding platform for Dicer [45] and other auxiliary factors involved in miRNA maturation [31], [42], [44]. We showed that RNA oligonucleotides targeting apical fragments of pre-miR-21, -33a and -210 affected their processing by Dicer (Figs. 2 and 3). However, we also observed that the RNA oligomer complementary to the terminal loop region of miR-16-1 precursor only weakly influenced the maturation of this miRNA (Fig. 3). This result is consistent with the observations of Michlewski et al., who reported that oligonucleotides targeting the terminal loop of miR-16-1 precursor (pri-miR-16-1) did not block the formation of mature miR-16-1. Michlewski et al. postulated that the processing of the miR-16-1 precursor was not affected by complementary oligonucleotides because it lacks a conserved structural element and consequently is not recognized by auxiliary factors. Our results indicate that the nucleotide composition of the apical fragment of the miR-16-1 precursor (AU-rich sequence) may be responsible for the observed effect. An AU-rich sequence cannot form a stable duplex with a 12-nt complementary nucleotide (Figs. 3 and 4). Thus, the latter cannot effectively inhibit the processing of this pre-miRNA. When we applied RNA oligomers that were complementary to the other partially single-stranded fragment of pre-miR-16-1, we observed a more efficient blocking of miR-16-1 maturation (Fig. 5). At present, any of these explanations cannot be unequivocally verified or falsified because the studies conducted by Michlewski et. al. concerned Drosha-mediated pri-miRNA processing in the nucleus, whereas our research focused on Dicer-mediated pre-miRNA cleavage, which occurs in the cytoplasm. Moreover, it has recently been shown that the same miRNA precursors may be targeted differently by nearly identical factors in different cell compartments. Piskounova et al. reported that although Lin28A and Lin28B selectively block the same let-7 pre-miRNA processing, and both have a high degree of sequence identity and conserved domain organization, they act through different mechanisms. Lin28A functions in the cytoplasm, and Lin28B operates in the nucleus [41]. We also found that the oligomers AL-21 and 2OMe-AL-16-1 affected pre-miRNA cleavage differently, even though both are capable of forming complexes with targeted pre-miRNA duplexes with very similar Tm values (Figs. 3B and 4A). These results might indicate that the Tm of the duplex formed by oligomer and miRNA precursor is not the only factor that affects the capacity of an oligomer to repress pre-miRNA processing by Dicer. Here, we show that the accessibility of miRNA precursors for binding with other molecules may also determine the level of miRNA production.

For the pre-miR-16-1/AL16-1_2 and pre-miR-21/AL-21 pairs, we observed that the oligomers similarly inhibited miRNA processing in both tested systems (commercial enzyme vs. extract). A different effect was observed for two other examined pairs, pre-miR-33a/AL-33a and pre-miR-210/AL-210. We observed markedly weaker inhibition of miRNA formation in experiments carried out in cytoplasmic extracts than in the corresponding experiments performed with commercial Dicer (Figs. 4A and 6). We also noticed that in reactions lacking an inhibitor (AL-33a or AL-210), the cleavage of the pre-miRNA was considerably less efficient in the extract system than in the commercial Dicer system. A similar observation was reported by Obernosterer et al., who postulated that specific inhibitory factors present in the cytoplasm might bind to individual miRNA precursors and block their conversion into mature miRNAs [28]. We apparently observed a similar phenomenon. An alternative hypothesis is that an unidentified factor interferes with the oligomer, preventing its binding with pre-miRNA. However, this cannot be the case because we also observed decreased miRNA processing in reaction mixtures lacking an inhibitor in the extract system.

**Figure 7:**
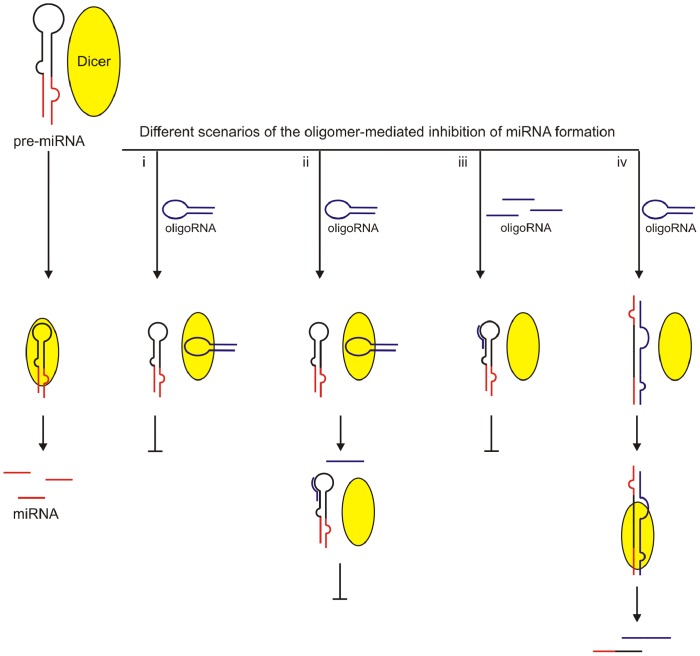
Proposed scenarios of short RNA-based regulation of miRNA production by Dicer. Schematic representation of a standard pre-miRNA cleavage by Dicer (*Left*). Four proposed scenarios (i–iv) of the oligoRNA-mediated regulation of miRNA processing by Dicer. Oligomers interacting with Dicer are presented as blue stem-loop structures, and oligomers that are not capable of interacting with Dicer are presented as short blue lines. A detailed description in the text.

Based on our observations, we can propose a new strategy for the precursor-specific regulation of miRNA processing by Dicer (Fig. 7). The key role in this strategy would be played by short RNA molecules that are capable of interacting with the enzyme, pre-miRNA, or both. The collected data indicate that miRNA processing can be regulated via several different scenarios. In the first scenario, the RNA oligomer functions as a typical competitor. It inhibits miRNA processing by interacting with a Dicer substrate-binding pocket. However, in this scenario the oligomer is not digested by Dicer (Fig. 7, i). In the second scenario, the oligomer also functions as a competitor, but in this case it is cleaved by the enzyme. In addition, one of the products of oligomer cleavage by Dicer binds to the pre-miRNA and precludes precursor digestion (Fig. 7, ii). In the third scenario, the application of very short oligomers disturbs the native structure of a miRNA precursor by interacting with its apical fragment. The resultant complex is not recognized and digested by Dicer (Fig. 7, iii). In the forth scenario, the oligomer also hybridizes with the precursor. In this case, however, the resultant complex is recognized by Dicer and cleaved but the pattern of precursor cleavage is altered and mature, functional miRNA is not formed (Fig. 7, iv). In all these cases, the complementarity-driven interactions are the key factor responsible for the specificity of the oligomer-mediated inhibition of miRNA formation.

In our experiments, we used *in vitro*-selected or artificially designed RNA molecules. Accordingly, our observations may not reflect the native conditions. There is, however, growing evidence that the cytoplasm contains a wide spectrum of short RNA molecules that hypothetically can interfere with miRNA processing. Recently, the discovery of 12-nt long RNA species corresponding to the 5′ regions of miRNAs, termed semi-miRNAs, has been reported [46]. These newly identified small regulatory RNAs were found to modulate the activity of the microRNAs from which they are derived. In addition, it is becoming increasingly evident that stable intermediates of RNA degradation can accumulate in the cell and function as signaling molecules or participate in mechanisms that control cellular pathways [47], [48], [49], [50]. However, more detailed studies are needed to confirm that these short RNAs influence miRNA biogenesis *in vivo*.

## Materials and Methods

### Cell Lines

Adherent HeLa cells (obtained from the American Type Culture Collection) were maintained in RPMI-1640 medium (GIBCO) supplemented with 10% fetal bovine serum (FBS), penicillin (100 U/mL), streptomycin (100 µg/mL), amphotericin B (250 ng/mL) and L-glutamine (2 mM).

### Oligonucleotides

All RNA molecules used in our studies were synthesized by Future Synthesis Sp. z.o.o. 2′-O-methylated RNAs contain modifications at every ribose. The sequences of all oligonucleotides are listed in Table S1.

### Oligonucleotide Labeling

The 5′-end labeling was performed using 10 pmoles of RNA, 1 µL γ-32P-ATP (3000 Ci/mmol, Hartman Analytic GmbH) and 10 U T4 polynucleotide kinase (Fermentas) in a final volume of 10 µL. After 10 min. of incubation at 37°C, the reaction was halted by adding 1 µL 0.5 M EDTA, pH 8.0. The radiolabeled oligonucleotides were PAGE-purified in 8% denaturing polyacrylamide gels and resuspended in water to a final concentration of approximately 10,000 cpm/ µL.

### Cellular Extract Preparation

HeLa cytosolic extracts were prepared as described by Leuschner et al. [33], with minor modifications. Approximately 8.4×10^6^ cells were harvested and collected by brief centrifugation at 100× *g* for 3 min. at room temperature. Then, the cells were washed once with 10 mL 1×PBS. Immediately after removing the PBS, the pellet was resuspended in 200 µL lysis buffer (30 mM HEPES, pH 7.4, 100 mM KCl, 5 mM MgCl_2_, 10% glycerol, 0.5 mM DTT, 0.2% NP40 and 1×Complete EDTA-free Protease Inhibitor Cocktail (Roche)) and incubated for 20 min. at 4°C with gentle agitation. To remove cell debris, extract was centrifuged at 700× *g* for 10 min. at 4°C and then at 13,000× *g* for 30 min. at 4°C. The supernatant (the cytosolic fraction) was transferred to a new tube, and the total protein concentration was determined using a Bradford assay (Bio-Rad). The extract concentration was subsequently adjusted to 10 mg/mL.

### Recombinant hDicer Cleavage Inhibition Assay

The Dicer cleavage assay was performed in 10 µL reactions containing 20 mM Tris-HCl, pH 7.5, 250 mM NaCl, and 2.5 mM MgCl_2_ buffer, 1 U human recombinant Dicer (Genlantis), pre-miRNA (containing 10,000 cpm labeled RNA) and the indicated oligonucleotide (with a molar ratio of pre-miRNA to oligonucleotide of 1∶1, 1∶10 or 1∶100). In addition, two control reactions were carried out: (i) a negative control (C−) with no enzyme and no inhibitor, to test the integrity of the substrate during the incubation time, and (ii) a positive control (C+) with enzyme but no inhibitor added. All samples were incubated at 37°C for 30 min. The reactions were halted by adding 1 volume of 8 M urea loading buffer and heating for 5 min. at 95°C and then separated on a 15% polyacrylamide/8 M urea gel. Data were collected using a Fujifilm FLA-5100 Fluorescent Image Analyzer and quantified using MultiGauge 3.0 (Fujifilm). The amounts of radiolabeled pre-miRNA and cleavage product/s were determined for each reaction, and the efficiency of miRNA production in the presence or absence of the oligomer was calculated to determine the oligomers’ capacity to inhibit pre-miRNA digestion by Dicer. The influence of the oligomers on miRNA production was expressed as a percentage, with the miRNA production in reactions lacking the oligomer defined as 100%.

### hDicer Cleavage Inhibition Assay in Cellular Extracts

The Dicer cleavage assay was performed in 10 µL reactions containing 20 mM Tris-HCl, pH 7.5, 50 mM NaCl, and 2.5 mM MgCl_2_ buffer, HeLa cytosolic extract (30 µg total protein), pre-miRNA (containing 10,000 cpm labeled RNA) and the indicated oligonucleotide (with a molar ratio of pre-miRNA to oligonucleotide of 1∶1, 1∶10 or 1∶100). In addition, two control reactions were carried out: (i) a negative control (C−) with reaction buffer but no extract and no inhibitor, and (ii) a positive control (C+) with extract but no inhibitor. All samples were incubated at 30°C for 1 hour. The reactions were halted by adding 1 volume of 8 M urea loading buffer and heating for 5 min. at 95°C and then separated on a 15% polyacrylamide/8 M urea gel. Data were collected using Fujifilm FLA-5100 Fluorescent Image Analyzer and quantified using MultiGauge 3.0 (Fujifilm). The amounts of radiolabeled pre-miRNA and cleavage product/s were determined as described above.

### pre-miRNA:RNA Oligomer Binding Assay

RNA complex formation was analyzed using an electrophoretic mobility shift assay (EMSA). The specific 5′-terminally ^32^P–labeled RNA oligonucleotide (1 pmol, 10,000 cpm) was mixed with the indicated pre-miRNA (100 pmol) in binding buffer (20 mM Tris-HCl, pH 7.5, 250 mM NaCl, 2.5 mM MgCl_2_), heated at 95°C for 3 min., and then slowly cooled to 37°C. In the control reaction, the pre-miRNA was not present. The samples were separated in 12% non-denaturing polyacrylamide gels supplemented with 5% glycerol at room temperature for approximately 6 hours at 7 V/cm in 1×TBE/5% glycerol buffer. The gels were exposed to a phosphorimager plate, which was subsequently scanned with Fujifilm FLA-5100 Fluorescent Image Analyzer to visualize the bands.

### Nitrocellulose Filter Binding Assay

The efficiency of ATD_13.6:pre-miR-210:hDicer complex formation was determined by filter binding assay on the Schleicher and Schuell dot blot apparatus [51]. A nitrocellulose membrane (Protran, Schleicher and Schuell) was pre-soaked in a reaction buffer containing 20 mM Tris-HCl, pH 7.5, 250 mM NaCl prior to use. The reaction mixtures contained the same concentrations of radiolabeled oligomer (10 pmol) and hDicer; only the amount of the substrate was changed. In each series, the following substrate:hDicer molar ratios were tested: 1∶1, 5∶1, 10∶1, 50∶1, 100∶1, 300∶1 and 600∶1. The control reaction lacked pre-miR-210. After the addition of pre-miR-210 to the mixture containing ATD_13.6 and hDicer, the reactions were incubated at 37°C for 30 min., and subsequently filtered on a nitrocellulose membrane. The amount of radioactivity retained on the filter was quantified using a Fujifilm FLA-5100 Fluorescent Image Analyzer.

### Bioinformatic Analyses

The secondary structures of RNA molecules were predicted with the RNAstructure 4.6 program (http://rna.urmc.rochester.edu/rnastructure.html). The melting temperatures were calculated using HyTher (http://ozone3.chem.wayne.edu) and RNA calculator (http://rnachemlab.ibch.poznan.pl).

## Supporting Information

Figure S1 **Binding of RNA oligomers to hDicer.** Reactions involving ATD_13.6 (**A**) and ATD_15.52 (**B**). hDicer was incubated with each RNA oligomer for 20 min. at 4°C. Nuclease activity of the hDicer was diminished by its 20 minutes’ preincubation at 4°C in a buffer lacking divalent metal cations. For each oligomer 5 reactions were carried out. One control reaction was run without hDicer (−). In the 4 other reactions hDicer concentration changed as follows: 0.034, 0.068, 0.136, and 0.272 mM. Reaction mixtures were separated in a native polyacrylamide gel. Triangles represent increasing amounts of hDicer.
(TIF)

Figure S2 **Binding of ATD_13.6 (A) and ATD_15.52 (B) to the selected pre-miRNAs.** Radiolabeled oligomer (either ATD_13.6 or ATD_15.52) was denatured and renatured alone (−) or in the presence of pre-miR-16-1, -21, -33a, and -210, respectively. The triangles indicate increasing amounts of pre-miRNAs (1, 10, and 100 pmoles). The reactions were separated in a native polyacrylamide gel. The position of the ATD_13.6/or ATD_15.52 and pre-miR-210 complex is indicated.
(TIF)

Figure S3 **The influence of increasing amounts of the selected pre-miRNAs on ATD_13.6 binding to Dicer.** Radiolabeled ATD_13.6 was incubated with hDicer and then the appropriate pre-miRNA was added (pre-miR-16-1, -21, -33a, and -210). Control reactions lacked the pre-miRNA (C+) or both pre-miRNA and hDicer (C−). Triangles represent increasing amounts of pre-miRNAs (the following oligomer:pre-miRNA molar ratios were applied: 1∶10, 1∶100, and 1∶300). The reactions were separated in a 5% native polyacrylamide gel. Positions of free ATD_13.6, the ATD_13.6:hDicer, and the ATD_13.6:pre-miR-210:hDicer complexes are indicated.
(TIF)

Figure S4 **The influence of 12-nt 2′-O-methylated oligomers on pre-miRNA processing by hDicer.** Each radiolabeled pre-miRNA (pre-miR-16-1, -21, -33a, or -210) was incubated with hDicer and 100 pmoles of the 12-nt 2′-O-methylated oligomer (2OMe-AL-16-1_2, 2OMe-AL-21, 2OMe-AL-33a, and 2OMe-AL-210), as indicated. In each reaction set, the oligomer targeting the apical fragment of the corresponding pre-miRNA hairpin, and the three non-matching oligomers were used. Control reactions lacked the enzyme and oligomer (C−) or the oligomer only (C+).
(TIF)

Figure S5 **Binding of 12-nt 2′-O-methylated oligomers to hDicer.** Reactions involving 2OMe-AL-16-1_2 (**A**), 2OMe-AL-21 (**B**), 2OMe-AL-33a (**C**), and 2OMe-AL-210 (**D**). Triangles represent increasing amounts of hDicer. The enzyme was incubated with each RNA oligomer for 20 min. at 4°C. Reaction mixtures were separated in a native polyacrylamide gel.
(TIF)

Table S1 **Sequences of all RNA molecules used in the study.**
(DOC)
