# Supplementary figures and images for: A New Short Oligonucleotide-Based Strategy for the Precursor-Specific Regulation of microRNA Processing by Dicer
Source: PLoS One. 2013 Oct 29;8(10):e77703. doi: 10.1371/journal.pone.0077703 (PMC3812226; doi:10.1371/journal.pone.0077703)

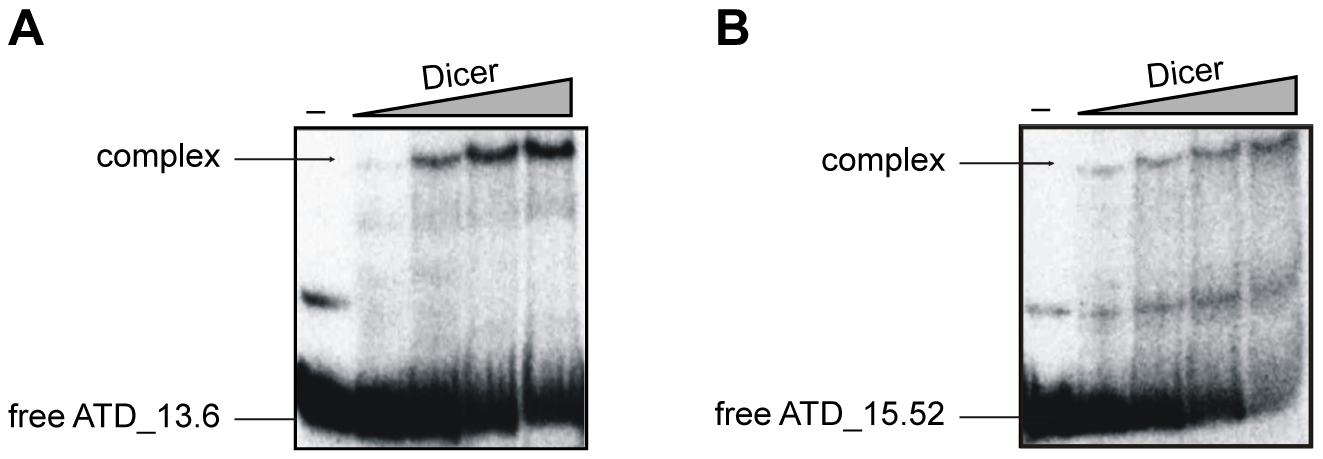

Supplement: Figure S1 — Binding of RNA oligomers to hDicer. Reactions involving ATD_13.6 (A) and ATD_15.52 (B). hDicer was incubated with each RNA oligomer for 20 min. at 4°C. Nuclease activity of the hDicer was diminished by its 20 minutes’ preincubation at 4°C in a buffer lacking divalent metal cations. For each oligomer 5 reactions were carried out. One control reaction was run without hDicer (−). In the 4 other reactions hDicer concentration changed as follows: 0.034, 0.068, 0.136, and 0.272 mM. Reaction mixtures were separated in a native polyacrylamide gel. Triangles represent increasing amounts of hDicer. (TIF) [file pone.0077703.s001.tif]

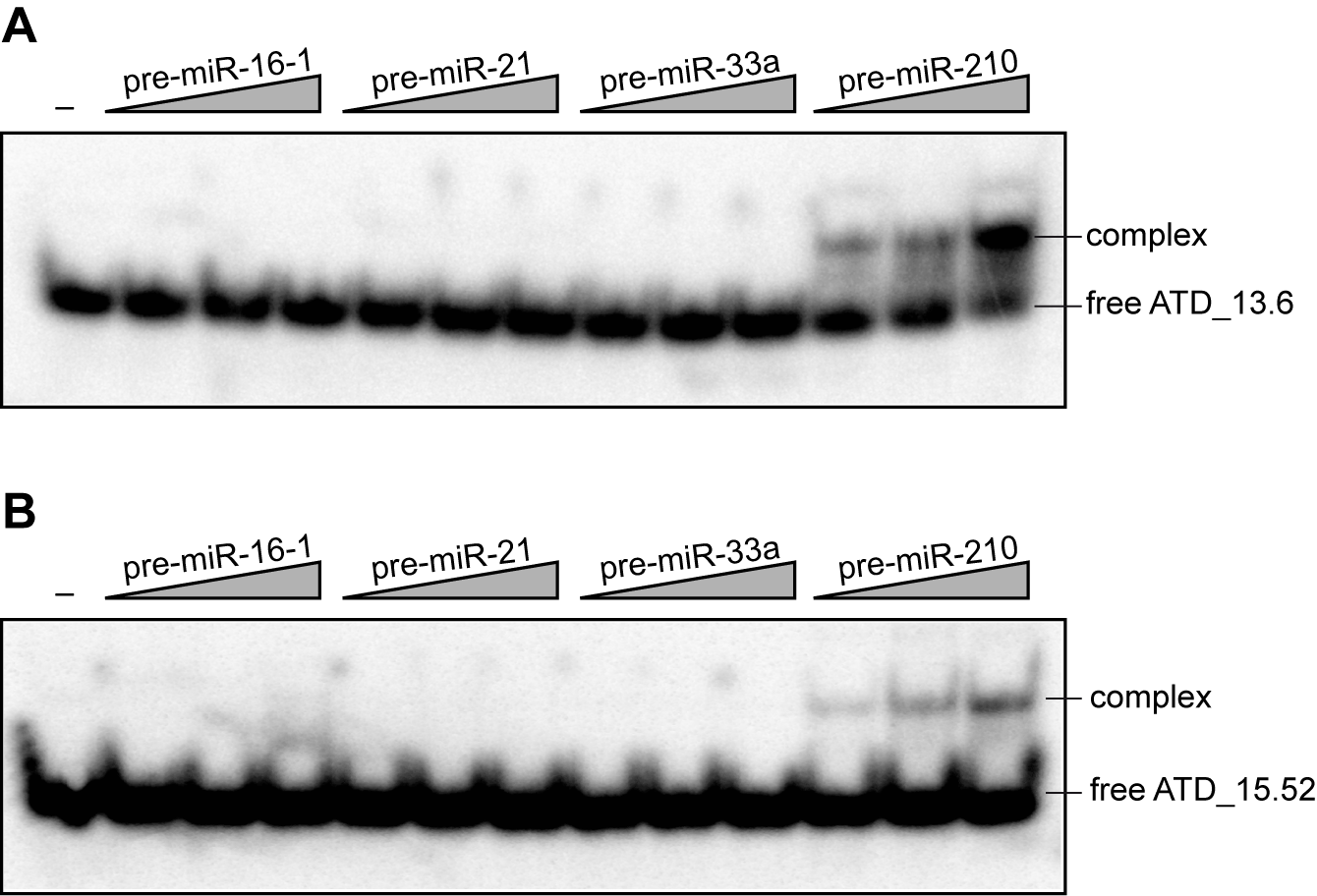

Supplement: Figure S2 — Binding of ATD_13.6 (A) and ATD_15.52 (B) to the selected pre-miRNAs. Radiolabeled oligomer (either ATD_13.6 or ATD_15.52) was denatured and renatured alone (−) or in the presence of pre-miR-16-1, -21, -33a, and -210, respectively. The triangles indicate increasing amounts of pre-miRNAs (1, 10, and 100 pmoles). The reactions were separated in a native polyacrylamide gel. The position of the ATD_13.6/or ATD_15.52 and pre-miR-210 complex is indicated. (TIF) [file pone.0077703.s002.tif]

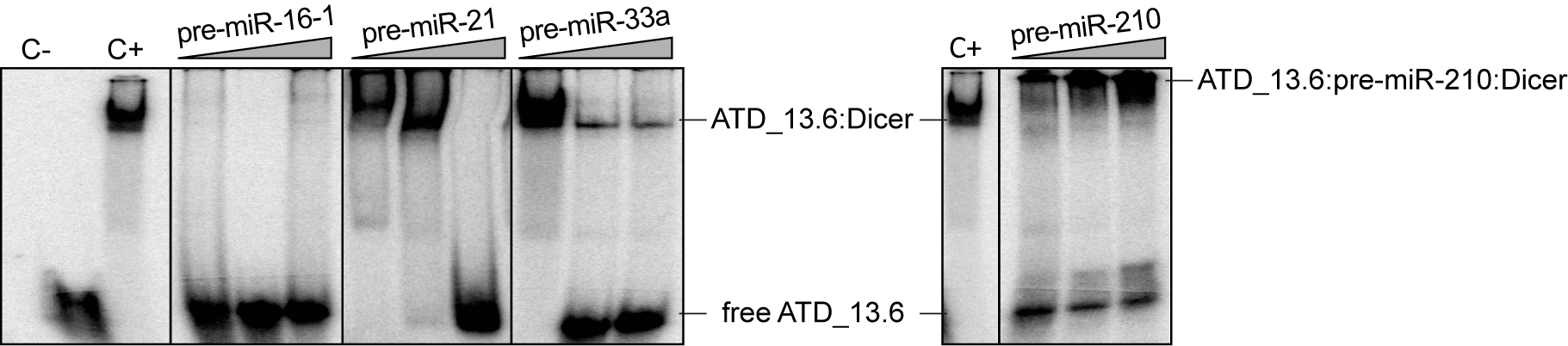

Supplement: Figure S3 — The influence of increasing amounts of the selected pre-miRNAs on ATD_13.6 binding to Dicer. Radiolabeled ATD_13.6 was incubated with hDicer and then the appropriate pre-miRNA was added (pre-miR-16-1, -21, -33a, and -210). Control reactions lacked the pre-miRNA (C+) or both pre-miRNA and hDicer (C−). Triangles represent increasing amounts of pre-miRNAs (the following oligomer:pre-miRNA molar ratios were applied: 1∶10, 1∶100, and 1∶300). The reactions were separated in a 5% native polyacrylamide gel. Positions of free ATD_13.6, the ATD_13.6:hDicer, and the ATD_13.6:pre-miR-210:hDicer complexes are indicated. (TIF) [file pone.0077703.s003.tif]

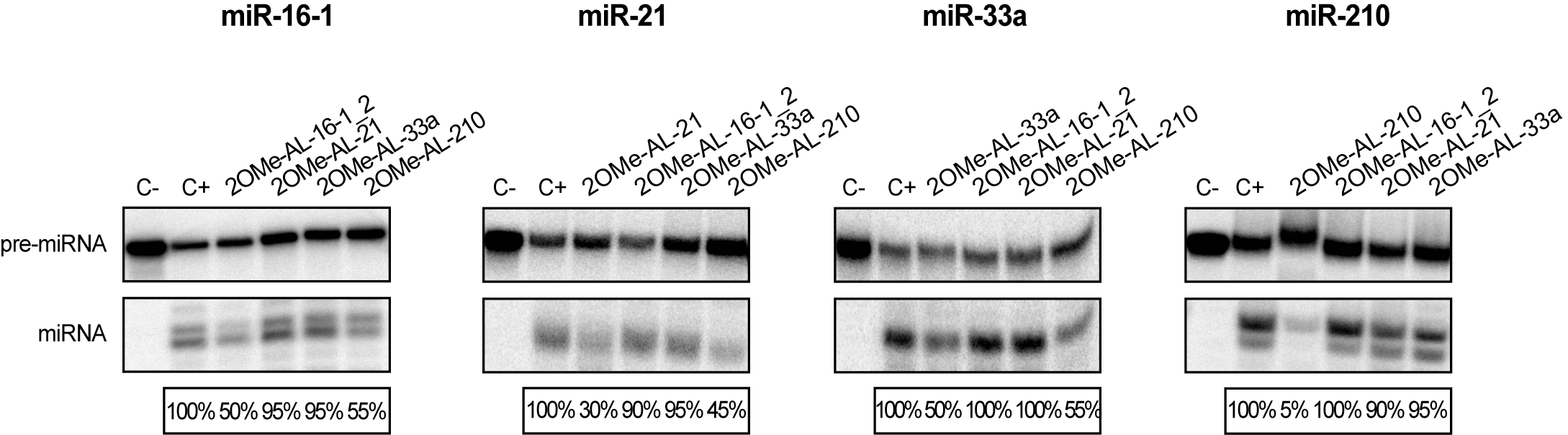

Supplement: Figure S4 — The influence of 12-nt 2′-O-methylated oligomers on pre-miRNA processing by hDicer. Each radiolabeled pre-miRNA (pre-miR-16-1, -21, -33a, or -210) was incubated with hDicer and 100 pmoles of the 12-nt 2′-O-methylated oligomer (2OMe-AL-16-1_2, 2OMe-AL-21, 2OMe-AL-33a, and 2OMe-AL-210), as indicated. In each reaction set, the oligomer targeting the apical fragment of the corresponding pre-miRNA hairpin, and the three non-matching oligomers were used. Control reactions lacked the enzyme and oligomer (C−) or the oligomer only (C+). (TIF) [file pone.0077703.s004.tif]

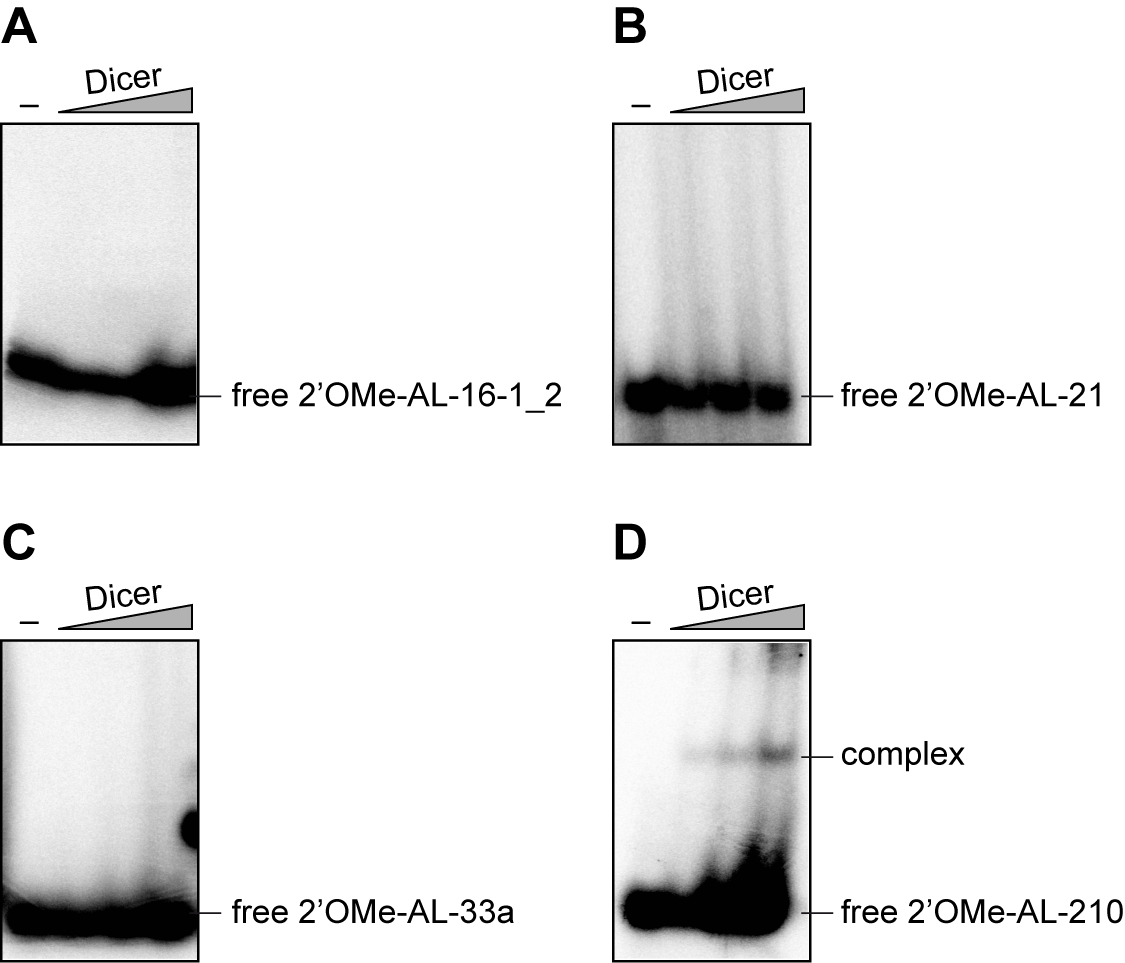

Supplement: Figure S5 — Binding of 12-nt 2′-O-methylated oligomers to hDicer. Reactions involving 2OMe-AL-16-1_2 (A), 2OMe-AL-21 (B), 2OMe-AL-33a (C), and 2OMe-AL-210 (D). Triangles represent increasing amounts of hDicer. The enzyme was incubated with each RNA oligomer for 20 min. at 4°C. Reaction mixtures were separated in a native polyacrylamide gel. (TIF) [file pone.0077703.s005.tif]
