# Supplementary material for: A New Short Oligonucleotide-Based Strategy for the Precursor-Specific Regulation of microRNA Processing by Dicer
Source: PLoS One. 2013 Oct 29;8(10):e77703. doi: 10.1371/journal.pone.0077703 (PMC3812226; doi:10.1371/journal.pone.0077703)
Supplement: Table S1 — Sequences of all RNA molecules used in the study. (DOC) [file pone.0077703.s006.doc]

**Table S1.** Sequences of all RNA molecules used in the study

| **NAME** | **SEQUENCE 5’->3’** |
| --- | --- |
| pre-miR-16-1 | UAGCAGCACGUAAAUAUUGGCGUUAAGAUUCUAAAAUUAUCUCCAGUAUUAACUGUGCUGCUGAA |
| pre-miR-21 | AGCUUAUCAGACUGAUGUUGACUGUUGAAUCUCAUGGCAACACCAGUCGAUGGGCUGU |
| pre-miR-33a | GUGCAUUGUAGUUGCAUUGCAUGUUCUGGUGGUACCCAUGCAAUGUUUCCACAGUGCAUC |
| pre-miR-210 | GCCCCUGCCCACCGCACACUGCGCUGCCCCAGACCCACUGUGCGUGUGACAGCGGCUG |
| AL-16-1/ 2OMe-AL-16-1* | AUUUUAGAAUCU |
| AL-21/ 2OMe-AL-21* | AUGAGAUUCAAC |
| AL-33a/ 2OMe-AL-33a* | GGGUACCACCAG |
| AL-210/ 2OMe-AL-210* | GGGGCAGCGCAG |
| 2OMe-AL-16-1_2* | GAAUCUUAACGC |

* RNA with every ribose 2’-O-methylated
